# A Journey of Dietary Therapies for Epilepsy in Iran: Diet Restriction in the Ancient Era to the Ketogenic Diet in the Modern Period

**Published:** 2019

**Authors:** Parvaneh KARIMZADEH, Aydin TABRIZI

**Affiliations:** 1 Department of Pediatric Neurology, Mofid Children’s Hospital, Faculty of Medicine, ShahidBeheshti University of Medical Sciences, Tehran, Iran; 2 Department of Pediatric Neurology, Pediatric Neurology Research Center, Research Institute for Children’s Health, Shahid Beheshti University of Medical Sciences, Tehran, Iran.

**Keywords:** Ketogenic Diet, Children, Diet Restriction, History

## Abstract

Epilepsy, in children, is a common neurological problem for referral to child neurology clinics.

The prevalence of nonfebrile seizure in children (under 10 years old), is estimated from 5.2 to 8.1 per 1000. Also, the prevalence of epilepsy in Iran estimated about 5 %; it means 4 million people of Iranian population live with epilepsy in Iran.

Although antiseizure drugs (ASDs) are the essential treatment modalities in most children, more than 30% of epileptic children have intractable seizures or they suffer from drug adverse effects secondary to these medications.

Because only a limited number of epileptic patients benefit from surgical therapy using the additional therapeutic options is inevitable. There are many available nonpharmacologic proven therapies for refractory seizures that Dietary therapy ( Ketogenic Diet) is one of the important therapeutic options in this group.

In this review, we will discuss the different features of pediatric epilepsy dietary therapies (Especially the Ketogenic Diet) in Iran and also the history of epilepsy in ancient Iran, utilization, effectiveness, side effects, tolerability, and acceptability as well as ongoing and future programs.

## Introduction

Epilepsy, a global health problem, is a brain cortex disorder characterized by an enduring predisposition to generate epileptic seizures in any age, sex and geographical regions without racism or socioeconomically class discrimination. It is also associated with a personal, familial, economical and psychosocial burden (1, 2). Up to 10% of the people worldwide experience at least one epileptic seizure during their normal lifespan (3). It is estimated that 50-70 million of world’s population live with epilepsy. According to the studies, approximately 80-90% of these patients live in developing countries such as Iran (4–7). 

In a meta-analysis and systematic review study, the prevalence of epilepsy in Iran estimated nearly 5 %.It means that 4 million epileptic people live in Iran, considering a total 80 million of population .The authors notified that the result is much higher than reported prevalence in other countries (8).

Although antiseizure drugs (ASDs) have proven efficacy in treatment of epilepsy, unfortunately they are not always effective. Approximately 30-33% of patients have drug resistant epilepsy (DRE) even when they are treated with multiple ASDs (9, 10). Current International League Against Epilepsy (ILAE) defines *Drug-resistant epilepsy *(also named *intractable epilepsy* or *refractory epilepsy*) as “failure of adequate trials of two tolerated, appropriately chosen and used antiepileptic drugs schedules (whether as monotherapy or in combination) to achieve sustained seizure freedom” (10, 11). DRE is a challenging aspect of children epilepsy treatment despite development of new generation of ASDs. Thus, a need for additional therapeutic options is inevitable (12, 13). There are many available nonpharmacologic proven therapies for DRE. Dietary treatments are one of these therapeutic options (14).

 In this review, we will address the different features of pediatric epilepsy dietary therapies in Iran including history of epilepsy in ancient Iran, utilization, effectiveness, side effects, tolerability, and acceptability as well as ongoing and future programs.

## Historical Perspectives

**The history of epilepsy**

**A) Epilepsy in the ancient world**

The history of epilepsy is as old as the history of humanity in the world (15).The stone tablets of the *Sakikku *(meaning “all diseases”), a Babylonian text compiled around 1000 BC, is the first known detailed description of various seizure types. The *Sakikku* refers to the epilepsy with the terms ‘antasubba’ and ‘miqtu’. The translated Babylonian text describes epilepsy features in the words such as unilateral and bilateral fits, the epileptic cry, the defecation incontinence, simple and complex epileptic seizures, the epileptic aura and narcolepsy (16).The Babylonians believed that epilepsy is secondary to supernatural powers and patients with epilepsy considered as a “person possessed by a particular demon or evil spirit” (17). The “falling sickness” is a translation of Sumerian term referring to descriptions of seizures (18).There is additional ancient reports about epilepsy descriptions including:

1- *“Hand of sin, god of the moon”* in *Mesopotamian* writings as *the**** Akkadian*** texts, dating back to 2000-4000 B.C.

2- *Loss of consciousness* in the ancient *Indian* medicine texts, dating back to 1500-4500 B.C.

3- The *Edwin Smith surgical papyrus,* ancient* Egyptian *medical texts, refers to epileptic convulsions in at least five cases in 1700 B.C.

4- The word ‘epilepsy’ originates etymologically from the Greek word “epilambanein”, which means ‘to seize, possess, or afflict and repeated attacks’. The disease was initially called sacred, because of the belief for its divine origin. *Heracletus of Ephesus* (535–475 B.C) referred the disease to the term *‘sacred disease’* with the hereditary nature, but not for describing epileptic seizures. The first formal description of epilepsy as a disease should be attributed to the *father of medicine*, *Hippocrates of Kos*, in his classic treatise on the* sacred disease *(19).

**B) Epilepsy in the ancient Iran**

The history of medicine in Iran is as old as the Persian civilization (20) when nearly 2700 years ago, two groups of Aryans, the Medes and the Persians, immigrated and settled in the Iranian plateau to the East of the Zagros Mountains and about two centuries later united and formed the Persian Empire (21).One of the earliest reports about epilepsy in Iranian people traced back to* Herodotus of Hallicarnassus* (484 - 425 B.C.), *the father of history*, in the third book of his work *The Histories (Thaleia).*He described in details the epileptic phenomena of the *Persian (Iranian) King Cambyses II, son of Cyrus. *According to* Herodotus*, King’s bizarre and aggressive behavior could be attributed to either the retribution of an aggrieved god or the so-called ‘sacred disease (22, 23). Generally, the medical history of ancient Persia and subsequently epilepsy related reports can be divided into three distinct periods.

***1-*** ***Avestan or Zoroastrian Medicine***

The Avesta, a collection of Zoroastrian holy writings, is the first Persian text that includes some documents of ancient Iranian medicine addressing the health and sickness. It was probably compiled during the 6th Century B.C., but the precise date is not determined. Apart from this Avestic reference, no other sources addressed to the Persian attitudes about epilepsy in those times (24, 25).

***2-*** ***The Sassanian Era***

The Persian king, Shapur I (242 to 272 AD) founded the city of Gondi-Shapur or Jundishapur on the site of present-day Ahwaz (26). Head of king Shapur II (Sasanian dynasty, 4th century CE) made Gondi-Shapur the capital of his empire (27). Gondi-Shapur became known as the ‘city of Hippocrates’ and the intellectual center of the Sasanian Empire. Its library contained 400 000 books and on its portal was engraved, ‘Knowledge and virtue are superior to sword and power’ (28). The academy of Gondi-Shapur offered training in medicine, philosophy, theology and science. Scholars from other countries studied different fields including medicine at the university (29). In this period, the Vendidad, the fourth section of the Avesta, script into which it was transcribed during the Sasan period, in the 6th Century A.C. (30). 

*3-* *Medieval* *Islamic Period*

In this period, epilepsy was one of important disease in the field of traditional Iranian neuroscience. The two major Iranian Hakims (practitioners), Muhammad ibn Zakariya al-Razi, known to the West as Rhazes (865-925 AD), and Ibn Sina, known as Avicenna (980-1037), considered epilepsy as a medical illness(31,32). "Qanoon fel teb of Avicenna or The Canon" and "Kitab al-Hawi of Razi orContinens" were reference texts in the western medical education from the 13th to the 18th centuries (33,34). In The Canon of Medicine (c. 1025), Avicenna described numerous mental conditions, including epilepsy, paralysis, stroke, vertigo, tremor, hallucination, nightmare, insomnia, mania, and dementia (35). Razi is considered as the *father of pediatrics* and *a pioneer of neurosurgery and ophthalmology*. In the "Continens", he described three cases of epilepsy treated by him (36).During the epileptic attacks, the patient fall to the ground, cry and froth at the mouth, Razi stated. In some patients, bladder or bowel incontinence may occur (37). 

Ibn Sina, the *“Prince of Doctors”,* was scientific and rational in his treatment of epilepsy. He was the first person to coin the term “epilepsy”, using a passive Latin verb. (38).With regard to epilepsy, Avicenna also adopted Galen’s theories, but he integrated them with some of his own original intuitions. In his *Canon of Medicine*, Ibn Sina describes epilepsy in comprehensive detail with its “variants and types along with clinical presentations and etiology”. He, also, offers therapeutic options for treatment of epilepsy .According to Avicenna, some of epilepsy features are following: 

1-The lower ventricle in the brain, is the exact origin of spasms because the initial presentations of epileptic attack involve the sense of sight and hearing as well as facial muscles and eyelids. 

2- Epileptic contractions are provoked by the presence of an unhealthy humor of which the body is trying to slough. 

3- Epileptic contractions are originated from the nerves in both longitudinal and latitudinal sense.

4- Melancholy is a more serious form of epilepsy with a distressing effect (10,39).

## The history of epilepsy dietary treatment

**A) Ancient period**

In the past, practitioners advocated many dietary treatments for epilepsy including either overuse or abstain from nourishments*. *However, fasting as a treatment for seizures was less recognized. Fasting is the only therapeutic measure against epilepsy recorded in the *Hippocratic collection*; complete abstinence from foods and drinks causes the effective cure of epilepsy. Fasting, as a therapy for seizures, was documented in biblical times. In a quotation from the King James Version of the Bible, Mark relates the story of Jesus curing an epileptic boy (15).

 The Iranian practitioners notified the importance of food and diet in treatment of epilepsy (31).One of these, Avicenna, suggested his theory on relationship between diseases and dietary regiment almost 1000 years ago: most illnesses are exclusively secondary to sustained mistakes of diet. This theory is a fundamental concept in the Islamic medicine. Based on ancient personalized medicine, he prescribed a long list of the herbal and pharmacological recipes and dietary rules for treatment of epilepsy (39, 41).In the Canon, some of his major consideration dedicated to treatment of epilepsy can be considered as the first ancient epilepsy guideline:

 1-The patients with epilepsy must avoid not only excessive eating but also some foods such as cow and sheep meat, fish, onion, garlic, celery, cauliflower, and carrot.

2-The patients may need surgery. The “operating therapeutic model” is being followed for centuries, not only in Iran but also throughout the world. 

3-Infants and young children with epilepsy should be protected from excessively shrill noises.

4-Epileptic children should not be exposed to intensely bright lights.

5- The patients with epilepsy must avoid hot and cold environments.

6- Extensive physical activity should be avoided either before or immediately after meals in epileptic patients. 

7-It is better that meals be arranged so that one eats three times a day.

8-The patients should eat goat meat instead of eating the meat of large mammals.He proposed that goat meat is much drier and thus reducing the process of “humidification” in the body. 

9-Celery eating, also, should be avoided because it excites the epilepsy, whereas coriander is beneficial because it inhibits the formation of vapors in the brain.10-He recommends abstinence from “fresh fruit due to its high water content; the heavier vegetables such as cabbage, turnips, and radishes; and any food that produces evaporations that could pass into the brain” (39-43).

**B) Modern period**

In Iran, the first epilepsy diet therapy center as a ketogenic diet established by Pediatric Neurology pioneer, *father of Iranian pediatric neurology*, Prof. Mohammad Ghofrani, in Shahid Beheshti University of Medical Sciences, Mofid Children Hospital in 1985 and it is more than 30 years that pediatric neurologists in Iran (especially in Mofid Children Hospital) administrated KD for children with DRE. Now, there are four major academic pediatric neurology departments in medical science universities of Shahid Beheshti, Tehran, Tabriz and Shiraz that have developed KD programs and clinics. These centers have been introduced as the focal points of “childhood epilepsy KD therapy” in the northwest of Iran (Tabriz center), south and southwest of Iran (Shiraz center) and remaining other regions of country (Shahid Beheshti and Tehran centers).Interestingly, epileptic patients from other countries such as Azerbaijan and Iraq referred to these centers to receive KD therapy, representing the significance of these focal points in offering services to neighborhood countries. Studies conducted in these centers have primarily like most similar studies focused on the effects of the KD on seizure control and its side effects (44).

## Ketogenic Diet

Parisian physicians, Gulep and Marie, introduced the first modern use of starvation as a modality for epilepsy therapy in 1911 .They treated 20 epileptic patients and reported the efficacy of starvation in reducing severity of seizures during treatment (43). In 1921, Dr. Russell Wilder suggested that the fasting induced ketonemia may reduce seizure frequency (45).Then, KD developed and practitioners have started to administration of KD for childhood DRE since 1924 (42). In 1930, Barborka treated 100 epileptic adult patients with the KD and reported seizure control during treatment, either completely or partially, in 56% of patients (46).The number of studies on KD has increasingly extended during past 40 years as more than 100 new KD related articles publish each year. Currently, there are four distinct types of KD available including (1) *the traditional “classic” KD*;(2) *the medium-chain triglyceride (MCT) diet*,(3) *the modified Atkins diet (MAD)*, and (4)*the low glycemic index treatment (LGIT)* (14).

## KD studies in Iran

In parallel with increasing use of the KD in the other countries, physicians in Iran have started to treat patients with the KD and subsequently several studies performed about “the role of KD in treatment of epilepsy” during past 30 years. We categorized these studies into four groups and evaluated them based on some results predominantly effectiveness, patients’ acceptance and side effects.

**1)** ***Classic KD studies***

Publications on classic ketogenic diet in either English or Persian (Farsi) languages were identified by systematically searching in online databases. A total of 8 published papers identified (Table 1). The first study that was classically conducted in Iran to determine the effectiveness of ketogenic diet in epileptic children refractory to medication, presented in “The 9th International Child Neurology Congress The 7th Asian and Oceanian Congress of Child Neurology” at September 20–25, 2002 Beijing, China by Prof. Mohammad Ghofrani, *founder of Pediatric Neurology in Iran*, and published in *Brain & Development Journal, Volume 24*(47). In this study, 215 epileptic children treated with KD at Mofid children’s hospital between 1989 and 1999. Mean age of the patients was 5.1 years (range: 2 to 12 years).The results were analyzed after starting classic KD for each patient at the end of first and sixth month. Among initial registered 215 patients 3, 23 and 92 patients left the study at the end of first, sixth and twelfth month, respectively.

**The results were as the following at the end of first month for 212 patients:**

*(1) Being seizure-free in 144 (67%)*

*(2) More than 50% reduction in seizure frequency in 22 (10.2%)*

*(3) Less than 50% reduction in seizure frequency in 16 (7.5%)*

*(4) No response to treatment in 30 (14%)*

***The results were as the following at the end of sixth month for 192 patients:***

*(1) Being seizure-free in 90 (41.8%)*

*(2) More than 50% reduction in seizure frequency in 32 (14.9%)*

*(3) Less than 50% reduction in seizure frequency in 22 (10.3%)*

*(4) No response to treatment in 48 (22.5%)*

**The results were as the following at the end of twelfth month for 123 patients:**

*(1) Being seizure-free in 21 (9.8%)*

*(2) More than 50% reduction in seizure frequency in 17 (17.7%)*

*(3) Less than 50% reduction in seizure frequency in 21 (9.7%)*

*(4) No response to treatment in 64 (31%)*

There was no statistically significant relationship between “response to treatment” and “follow up time”, but there was significant difference between “response to treatment” and “reduced number of ASDs” (48).

**Later, other neurologists performed several researches in the field of classic KD along with other types of KD in the all age groups of patients with epilepsy (Tables 1, 2 and 3).**

In one study, 87 patients with epilepsy treated with KD at Mofid children’s hospital between 1999 and 2006 to evaluate the efficacy and acceptability of the classic ketogenic diet. Mean follow-up time was 9.4 ± 9.8 SD (range 1-24 months). Only children who had DRE or who had severe adversereactions to ASDs were entered to the study. On the first day of admission, paraclinic investigations such as EEG and laboratory studies were carried out. All sugar-containing medications were discontinued. Then, the fasting initiated in the hospital and continued for 24-72 hours when 4+ ketonuria was detected in urine analysis. Later, the diet was started at the classic 4:1 ratio (ratio of grams of fat to protein plus carbohydrate).Younger children were started at the lower ratio (3:1) to allow more protein intake. Fluids were restricted to 75% of maintenance level*.* Total carbohydrate intake was not more than 30 gram andnot less than 10 gram per day. Prior to discharge, mothers had been educated, supplements were added and ASDs were reduced. Patients followed up at hospital KD clinic at 1, 3 and 6 months after discharge. At the end of the first month,seizure frequency reduced to at least 50% in 71% of patients. At the end of the third month, acceptable reduction in seizure frequency (at least 50%) occurred in 87% of children, of which 39% had complete seizure control. At the end of sixth month, seizure frequency reduction continued in 63% of patients (49).This results was identical to the previous study in Classic KD in Iran (48).

In another study, 66 children with DRE treated with classic KD from October 2008 to June 2010.Inclusion criteria were as following: 

*1) Age between 1and16 years old2) No neurodegenerative disorders and neoplasm of CNS3) No febrile convulsions4) the acceptable literacy of parents (at least school education).*In the first meeting, the trained dietitian explained the ketogenic diet and the goals of the study to the family. Then, the parents signed informed consent forms and before starting the diet, parents were asked to register the frequency of their child’s seizures (number of seizures/day) for a period of one week. The patients were hospitalized in the pediatric neurology ward. Fasting and fluid restriction was undertaken when 3+ to 4+ ketonuria was detected. The patients were then fed a single daily meal of a 3:1 ratio. The diet gradually increased in amount to provide the total daily required calories. High-fat foods provided one third of the total daily required calories on the first day, two thirds of the required calories on the second day and full required calories on the third day of treatment. Efficacy was defined as a reduction of more than 50% in seizure frequency. After 3 months, 80% of the patients kept the diet. After one week, one month and 3 months, there was a more than 50% reduction in the frequency of the seizures in 40 (60%), 50 (75%) and 39 (59%) patients, respectively. Three patients (4.5%) were seizure-free after 1 week, 12 (18%) were seizure-free after one month and 12 (18%) were seizure-free after three months (50).

An open label trial was done to evaluate the effects of KD on serum lipid profile. In this study, 33 children with DRE treated with the KD and were followed for 6 months. Their serum lipid profile was assessed at baseline. After 6 months of administering the diet, median serum levels of triglyceride (TG), total cholesterol (Chol), low density lipoprotein (LDL) and high-density lipoprotein (HDL) were significantly increased. However, at 3 and 6 months after initiating the diet, seizure frequency was reduced in 63 % of children. Results of this study indicated that a classic KD in children with refractory seizures is effective in seizure reduction, but leads to development of hypercholesterolemia and hypertriglyceridemia (51).

Another prospective, open-label study was carried out to investigate the influence of the KD on liver function. A total of 28 children and adolescents with DRE who did not respond to at least 2 ASDs enrolled the study but half of them left the study. Liver function tests including Alanine aminotransferase (ALT), Aspartate aminotransferase (AST), and alkaline phosphatase (ALP) were measured before and after 3 months of starting KD. The serum levels of ALP and AST did not differ significantly before and after 3 months on the diet, while a significant difference identified in the serum level of ALT before and after 3 months of treatment (52). 

In a study, the KD effect on the growth of 14 girls and 22 boys with DRE evaluated .Mean age of the patients was 4.1 years (range: 2 to 7 years).Comparison of the studied parameters before the initiation of the diet and 3 months later showed significantly reduction in weight as well as serum levels of hemoglobin, calcium, glucose. Moreover, mean height growth velocity of 2.5cm per year was lower than normal growth velocity chart for age. In conclusion, the results showed that although the patients’ height growth velocity did not statistically change, their mean weight, and mean serum levels of hemoglobin, calcium, and glucose decreased significantly. The study, also, showed significant increase in the serum levels of Chol, TG, VLDL, and LDL/HDL and Chol/HDL (53).

In another study, 43 children with DRE treated with classic KD between 2003 and 2005.At the end of first, third, sixth and twelfth month, 50% reduction in seizure frequency were observed in 77%, 65%, 44% and 35% of patients (54).

In another open label trial, to evaluate the efficacy, safety, and tolerability ofa classic 4:1 ketogenic diet using a formula-based power (Ketocal), 27 infants and children with DRE and aged between 12 months and 5 years entered to the study. Of 27 children, 5 were lost to follow-up and 22 were remained at the end of the study. None of these children discontinued the diet because of the complications. Thirteen children and their parents (59%) reported that the diet was palatable andtolerable enough. After 4 months, the median frequency of seizures per week reduced more than 50% and 90% in 15 (68.2%) and 6 (27.3%) children, respectively. The authors concluded that the ketogenic diet using a formula-basedpowder (Ketocal) is effective, safe, and tolerable in infants and children with DRE who are reluctant to eat homemade foods (55).

**2)** ***Modified Atkins Diet (MAD) studies***

A total of 3 published papers identified regarding modified Atkins diet by systematically searching in online databases (**Table 2**).A clinical trial study was conducted in 51 epileptic children aged 1 –16 years with DRE from 2004 to 2006.Among patients,27 left the study for various reasons and remaining 24 continued the Atkins diet for a minimum of three months. Carbohydrates were initially limited to 10 g/day and fats constituted 60% of the total energy requirement. Following three months of treatment with the Atkins diet, 16 patients (67%) had more than 50% reduction in seizure frequency, and 6(25%) had more than 90% improvement, of whom 5 were seizure-free. Mean seizure frequency after the first, second and third months of treatment were significantly lower than at baseline. Results showed that the Atkins diet can be considered as a safe and effective alternative therapy for childhood DRE .Atkins diet was well tolerated in patients. No serious complications were observed during the trial; however, in some of the 27 patients who left the study, minor side effects such as nausea, vomiting, and loss of appetite were the probable causes of diet discontinuation (56).

In another study, 21 children in the age range of 4-17 years with DRE were treated with the Modified Atkins Diet prospectively in a hospital based ambulatory clinic between 2006 and 2008. These children were experiencing more than four seizures per month which could not be controlled by any combination of three or more ASDs. Patients with evidence of metabolic disorders were excluded. Efficacy was evaluated based on the comparison between the average monthly baseline seizure frequency (3 months before diet initiation) and seizure frequency after 6 months of starting MAD. Six months after initiating the modified Atkins Diet, 52.4% of the patients remained on the diet and 42.9% experienced more than 50% reduction in seizures (57).

In a controlled randomized clinical trial, 66 refractory adult epileptic cases treated with MAD from 2010 to r 2012.They were randomly dividedinto two groups:

1) A case group (22 patients): a group that treated with both ASDs and MAD. 

2) A control group (32 patients): a group that treated only with ASDs.

 The primary outcome was at least 50% reduction in seizure frequency after 2 months of therapy. Also, the study showed that MAD co-therapy in case group can decrease seizure frequency 2.19 times in comparison with control group. No significant difference was shown between groups regarding baseline characteristic (58).

***3) Low Glycemic Index Diet (LGID) studies***

In searching of online databases, only one published article found regarding Low Glycemic Index Diet (LGID).In this study as a first Middle East report about LGID, 42 children with DRE, aged between 1.5 and 17 years treated with LGID from 2009 to 2011. LGIT was initiated on an outpatient basis. The diet was composed of 65% fat, 25% protein and 10% carbohydrate (40–60 g), and the glycemic index of foods was limited to below 50. At the study entry, 84% of patients were categorized as having more than one seizure per day, with the remaining children as experiencing over one seizure per week. A more than 50% seizure reduction was observed in 71.4% of the patients after the second week, in 73.8% at the end of the first month and in 77.8% at the end of the second month. In 30% of the patients a mild increase in blood urea nitrogen (BUN) was detected. The ratio of fat to carbohydrate and protein grams was 1:1 with less fat compared to MAD and classic KD. Treatment efficacy is not related to the levels of ketone yet correlates with lower and steadier blood glucose levels. In this study, LGIT had fewer side effects when compared to KD, a characteristic that is of great value for young children. The study showed 12.6% increase in serum triglycerides levels without significant metabolic implication. Although elevated BUN levels detected in approximately one-third of patients that were probably the result of protein intake, there is likely no relationship between excessive protein intake and kidney failure in normal subjects. Therefore, it is recommended that LGIT patients followed closely up for renal function, higher fluid intake, and modifications in protein intake if necessary. In conclusion, this study indicated some benefits for LGIT as following:

*(1) High compliance and better tolerability compared with classic KD(* *2)* *LGIT is a safe and effective adjuvant antiepileptic therapy and may be used as an alternative to the classic KD in conditions when this diet cannot be used for any reason(3) Easy preparation without detailed meal plans and weighing food on a gram scale *

*(4) Improved palatability because of more liberalized carbohydrate content and decreased fat content*

*(* *5) F* *ewer psychosocial issues as seen in classic KD*

*(6) Ability of the patients to eat outside the home without the need to prepare special meals.*

*(7) The low cost of the outpatient implementation of LGIT was a main benefit (* 59 *).*

**4)** ***Comparative KD studies***

Two papers from Iran have been published to date regarding comparison of efficacy of KD types with each other or with non dietary treatments (**Table 3**). A clinical trial study performed on 40 children with DRE between 2005 and 2010.The subjects categorized into two groups. Initially, 20 patients in first group were treated with the Atkins diet from January 2005 to October 2007, and then 20 patients in the second group treated with the classic KD from October 2007 to March 2010. At the end of first, second, and third month, more than 50% reduction in seizures frequency occurred in 55%, 30%, and 70% of patients in the KD group and in 50%, 65%, and 70% of cases in the Atkins diet group, respectively. The study showed there was no significant difference between the classic Ketogenic diet and the Atkins diet at the end of first, second, and third month and both groups had similar responses to the treatment (60).

In another study, 40 children with DRE were treated with the classic KD and IVIG in 1995. The subjects categorized into two groups. The first group treated with classic KD and the second group treated with IVIG. Seizure frequency reduced 64% and 52% in the first and second group at the end of sixth month, respectively. However, difference between two groups was not significant (61).

## Discussion

**A)** **The basic epidemiologic data and optimized epilepsy care services**

***1) Why the basic epidemiologic studies are important?***

Epidemiologic studies conducted regarding to the prevalence of epilepsy are more than those of incidence in developing countries. Moreover, it is generally reported that epilepsy is more prevalent in developing than in developed countries (62, 63). During the last several decades of the 20th century, plenty of studies have been done to determine the prevalence of epilepsy in developing countries and children (64, 65). The prevalence of epilepsy in developed countries ranges from 4 to 10 cases per 1000 (66).However, higher prevalence of epilepsy, ranging from 14 to 57 cases per 1000, have been reported in the developing and tropical countries. Higher prevalence of epilepsy in the these countries is probably due in part to differences in study methodology, problems with case ascertainment, lack of standardized classification, and poor epilepsy diagnostic accuracy tools (67, 68). On the other hand, a variety of epidemiological studies of the prevalence of epilepsy in children have been done in many countries. In the most studies, prevalence reported 4 to 5 per 1,000 children, or about 0.5 percent. Also, studies indicated that the prevalence increases with age, ranging from approximately 2-3/1000 to 4-6/1000 in 7 and 11-15 year olds, respectively (65).

Because epilepsy is a heterogeneous disorder, study of simple prevalence and incidence do not demonstrate many aspects of the condition’s characteristics including “response to variety of pharmacological and non pharmacological treatments” in a general population (68). Besides the incidence and prevalence, epidemiological data provide knowledge about and potential risk factors for epilepsy, and can be used for planning health services including development of “organized epilepsy unites” in the hospitals, especially “KD clinics” for people with DRE(32).

According to the WHO, nearly 80% of the people with epilepsy living in developing countries receive no treatment at all (69).In a review performed to the evaluation of a proportion of epileptic patients receive no care ( a “treatment gap” ), demonstrated that 75% of epileptic people with treatment gap live in low-income countries(70). As a result, they expected to have more “epilepsy induced morbidity and psychosocial consequences”, while in the most of them completely control of epilepsy is not impossible and they can return to a normal life by taking an appropriate care provided there were an essential epidemiologic data (71).

***2) How many national epidemiologic studies have been done for epilepsy in Iran?***

In searching of online databases, there were a few published articles regarding national epidemiology of epilepsy in Iran. In a study with population size of 35014, estimated crude prevalence (per 100 persons) was 12 (72).In another epidemiologic study, the prevalence of epilepsy was 1.8% and it was more common in females, unemployed people and higher educational level. Authors concluded that the lifetime prevalence of epilepsy in Iran is not low (73). In a meta-analysis and systematic review study, the prevalence of epilepsy in Iran estimated nearly 5%, a result that surprisingly was much higher than reported prevalence in other countries (8).

**3)** ***What is the prevalence of epilepsy in neighborhood and similar countries to Iran?***

Asian studies documented a prevalence of 2.2 per 1000 persons in India (1,74-76). The crude prevalence rate of epilepsy was 9.98 per 1000 persons in Pakistanand 7.0 per 1000 persons in Turkey. In both countries the prevalence was roughly twice as high in rural compared to urban areas (12,77). Lifetime prevalence of 7.0 per 1000 persons has been reported in China (78). An approximate724,500 people with epilepsy live in the Arab world. An incidence of 174 per 100,000 persons in 2001 was reported in a hospital-based study from Qatar. Prevalence ranged between 0.9/1,000 in Sudan and 6.5/1,000 in Saudi Arabia, with a median of 2.3/1,000. All the studies report higher prevalence in males, which was statistically significant in the Saudi study (79). 

The incidence of epilepsy in children ranges from 41- incidence is consistently reported to be highest in the first year of life and declines to adult levels by the end of the first decade. The prevalence of epilepsy in children is consistently 187/100,000. Higher incidence is reported from underdeveloped countries. The incidence is higher than and ranges from 3.2-5.5/1,000 in developed countries and 3.6-44/1,000 in underdeveloped countries. Either prevalence or incidence seems highest in rural areas (79 - 81).

**3)** ***What is the conclusion?***

In almost all the Middle East countries, mental health units in ministries of health are also planning and implementing for epilepsy. In the Islamic Republic of Iran, the health information system, which includes mental disorders, also encompasses epilepsy (32).Therefore, in order to develop national and adapted models for the promotion of epilepsy control including initiation of advanced epilepsy centers with KD clinics and reduce the treatment gap and social and physical burden, it is important that Iranian ministry of health, neurology associations, academic university experts and other related practitioners cooperate to do essential studies especially epidemiologic ones and subsequently use their results information to planning epilepsy care services.

**B) Comparison of ketogenic diets’ studies between Iran and other countries**

As tables 1, 2 and 3 showed, a total of 14 researches have been done in the field of KD and epilepsy – most of which (57%) are about classic KD- since 1989.In these studies, researchers evaluated several features of KD including effects, tolerability, side effects and acceptance. Also, as mentioned earlier, there are four active KD clinics in Iran offering services to children with DRE, while more than 150 centers outside of the USA and 100 centers inside the USA(83) provide the ketogenic diet programs (81, 82).In this review, approximately all Iranian studies reported that KD, either classic KD or MAD and LGID, were effective in treatment of epileptic children and adults with minimal side effects. With availability of Medline in 1965, more than 900 human studies of dietary therapies for epilepsy have been published to date (83). Approximately 100 new articles concerning epilepsy dietary therapy are being published each year. Although initiation and management of the KD around the world are different, the benefits are similar (84). Hundreds of studies over the past two decades have shown significantly results identical to Iranian studies (14).In almost all studies, a conclusion have been similar: approximately 50–60% of children will have at least more than 50% seizure reduction, with one third have more than 90% response (85-87).

Several studies with retrospective, prospective and Meta analyses methodologies have indicated the efficacy of KD in intractable childhood epilepsies (88 -92). In a Cochrane review based Meta analyses of four randomized controlled studies (93-96), the efficacy of KD on epilepsy was confirmed in 289 children and adolescents (97). A meta-analysis of 19 studies including a total of 1084 children showed more than 90% seizure reduction in one-third of patients and more than 50% seizure reduction in half of the patients (92).A large prospective KD study including 150 children with DRE, represented 50-90% seizure reduction in 26% of children and 90% seizure reduction in 31% of children (98). A Chinese KD study including 317 children with DRE, showed more than 50% seizure reduction in 35%, 26.2%, and 18.6% of children after 3, 6 and 12 months, respectively (99). 

A recent meta-analysis of studies from 1925 to 1998 reports that 30% of patients have a 50% to 90% reduction in seizures and an additional 37% of patients have more than a 90% reduction in seizures (100).

In an Indian prospective study of 27 children with DRE, more than 50%seizure reduction occurred in 48% of patients at 6 months and, more than 50% seizure reduction occurred in 37% of patients at 12 months (101). KD is shown to be more effective than most ASDs and to reduce seizure frequency at least by 50% in half of the patients (102).Also, it is shown that recurrence rate of seizures is reduced after discontinuation of KD (103). It seems that efficacy with the KD does not decrease over the years, and seizure control continues many years later, surprisingly even after the KD has been discontinued (104,105). 

The first formal, prospective, open-label study of MAD was designed and 20 children with DRE were treated (106).Also, the benefits of the MAD confirmed in two studies from Korea (107,108).

In a randomized controlled study of the MAD for the treatment of childhood epilepsy, the likehood of more than 50% and 90% seizure reduction was higher compared to the control group of standard medical management (109). In a study from Korea, 14 children with DRE evaluated in terms of the efficacy, safety, and tolerability of MAD. At 6 months, more than 50% seizure reduction occur in patients (106).

The only study from Iran suggested that LGIT could reduce seizure frequency in alarge number of patients with DRE (60). The first study of LGIT was published in 2005 with promising results. In a study, authors reported more than 50% reduction in up to 66% of the patients compared to baseline seizure frequency after 12 months (110).
